# A qualitative study to understand guideline-discordant use of imaging to stage incident prostate cancer

**DOI:** 10.1186/s13012-016-0484-5

**Published:** 2016-09-02

**Authors:** Danil V. Makarov, Erica Sedlander, R. Scott Braithwaite, Scott E. Sherman, Steven Zeliadt, Cary P. Gross, Caitlin Curnyn, Michele Shedlin

**Affiliations:** 1 VA New York Harbor Healthcare System, 423 E 23rd St, New York, NY USA; 2 Department of Urology, NYU Langone Medical Center, 150 E 32nd St, New York, NY USA; 3 Department of Population Health, NYU Langone Medical Center, 550 First Avenue, TRB, New York, NY USA; 4 VA Puget Sound Healthcare System, 1600 S Columbian Way, Seattle, WA USA; 5 Department of Internal Medicine, Yale School of Medicine, E.S. Harkness Memorial Hall, 367 Cedar Street, New Haven, CT USA; 6 NYU College of Nursing, 433 First Avenue, New York, NY USA

**Keywords:** Theoretical domains framework, Prostate cancer, Imaging, Guideline, Qualitative, Semi-structured interviews

## Abstract

**Background:**

Approximately half of veterans with low-risk prostate cancer receive guideline-discordant imaging. Our objective was to identify and describe (1) physician knowledge, attitudes, and practices related to the use of imaging to stage prostate cancer, (2) patient attitudes and behaviors related to use of imaging, and (3) to compare responses across three VA medical centers (VAMCs).

**Methods:**

A qualitative approach was used to explore patient and provider knowledge and behaviors relating to the use of imaging. We conducted 39 semi-structured interviews total—including 22 interviews with patients with newly diagnosed with prostate cancer and 17 interviews with physicians caring for them—between September 2014 and July 2015 at three VAMCs representing a spectrum of inappropriate imaging rates. After core theoretical concepts were identified, the Theoretical Domains Framework (TDF) was selected to explore linkages between themes within the dataset and existing domains within the framework. Interviews were audio-recorded, transcribed verbatim, and then coded and analyzed using Nvivo software.

**Results:**

Themes from patient interviews were categorized within four TDF domains. Patients reported little interest in staging as compared to disease treatment (*goals*), and many could not remember if they had imaging at all (*knowledge*). Patients tended to trust their doctor to make decisions about appropriate tests (*beliefs about capabilities*). Some patients expressed a minor concern for radiation exposure, but anxiety about cancer outcomes outweighed these fears (*emotion*). Themes from physician interviews were categorized within five TDF domains. Most physicians self-reported that they know and trust imaging guidelines (*knowledge*) yet some were still likely to follow their own intuition, whether due to clinical suspicion or years of experience (*beliefs about capabilities*). Additionally, physicians reported that medico-legal concerns, fear of missing associated diagnoses (*beliefs about consequences*), influence from colleagues who image frequently (*social influences*), and the facility where they practice influences rates of imaging (*environmental context*).

**Conclusions:**

Interviews with patients and physicians suggest that physicians are the primary (and in some cases only) decision-makers regarding staging imaging for prostate cancer. This finding suggests a physician-targeted intervention may be the most effective strategy to improve guideline-concordant prostate cancer imaging.

## Background

The widespread use of prostate-specific antigen (PSA) screening has led to prostate cancer stage migration and changing paradigms of disease management [1, 2]. Contemporary patients are unlikely to have advanced disease, decreasing the clinical utility of diagnostic imaging in their staging evaluation. Professional societies and policy organizations have been unanimous in their prostate cancer imaging guidelines and quality measures: imaging should be reserved for patients with high-risk disease and low-risk patients should not undergo imaging [3–9]. For example, the National Comprehensive Cancer Network (NCCN) Guidelines for Prostate Cancer include precise recommendations for which prostate cancer patients require advanced imaging prior to treatment [7, 10]. These patients should have a life expectancy greater than 5 years or demonstrate symptoms of metastatic disease (e.g., bone pain, hematuria, weight loss). Pelvic computerized tomography (CT) or magnetic resonance imaging (MRI) should be used for those patients with locally advanced (T3) or metastatic (T4) cancer or those with clinically localized (T1–T2) disease should their probability of lymph node involvement exceed 10 %. Radionuclide bone scan is recommended only for T1 patients with PSAs greater than 20 ng/mL, T2 patients with PSA greater than 10 ng/mL, Gleason score greater than 7, locally advanced or metastatic cancer, or symptoms suggesting bone metastases. No other patients require staging imaging.

In spite of these clear proscriptive recommendations, guideline-discordant imaging is common: almost half of men with low-risk, localized prostate cancer receive guideline-discordant, unnecessary imaging. Inappropriate prostate cancer imaging was named by the American Society of Clinical Oncology (ASCO) and the American Urological Association (AUA) among their top priorities for *Choosing Wisely*, a nation-wide initiative to encourage stewardship of medical resources [11, 12]. Imaging rates among men with low-risk prostate cancer have been reported to be as high as 19–74 % in a community cohort, 10–48 % in a SEER-Medicare cohort, and 41 % in the Veterans Health Administration (VHA) [13–16]. There is a simultaneous underuse of imaging among men with high-risk disease. Men with high-risk cancer from SEER-Medicare only underwent bone scan 70–75 % of the time and only 57–58 % underwent CT, for a total rate of 66 % receiving guideline-concordant, appropriate imaging; within VHA, only 70 % receive appropriate imaging for high-risk prostate cancer [15–17]. The patterns of guideline-discordant imaging in VHA are particularly surprising given the absence of the types of financial incentives driving utilization in the fee-for-service setting [14]. Retrospective studies have suggested that non-clinical factors could be creating a barrier to guideline-concordant imaging in VHA. For example, veterans with low-risk prostate cancer who were dual users of VHA and Medicare had higher odds of inappropriate imaging as compared to those using VHA benefits only, while dual users with high-risk cancer had similar odds of appropriate imaging. This finding suggests that health system or insurance status plays a role in the decision to image men with prostate cancer [18]. Additionally, clinical factors (i.e., Gleason score, PSA, and clinical stage) are associated with imaging utilization in guideline-discordant ways [7, 16, 18–22]. Perhaps because physicians rely on personal imaging heuristics, rather than on strict interpretation of guidelines, prior quality improvement efforts seeking to eradicate inappropriate imaging also had a negative effect on *appropriate* imaging, limiting utilization of a guideline-indicated procedure [4, 15, 22, 23]. The causes of these behavioral modifications are perplexing at least in part because so little is known about the reasons why physicians and patients utilize or do not utilize imaging to stage prostate cancer.

We sought to explore the use of prostate cancer imaging using a qualitative research approach. Qualitative studies in this topical area that seek to further understand guideline rejection aim to identify barriers and facilitators to physician/health care provider adherence. Yet, to our knowledge, there have been no efforts to understand the practice of prostate cancer imaging using qualitative methods [24, 25]. Our findings will be of great interest to patients, physicians, policy makers, and those seeking to improve the quality of care for men with prostate cancer.

## Methods

### Design

We conducted semi-structured interviews with both prostate cancer patients and physicians who treat patients with prostate cancer at three VA medical centers (VAMCs). Originally, Grounded Theory was used to allow for categories to emerge organically during interviews. However, after conducting and analyzing several physician and patient interviews, the research team observed that the emerging categories clearly aligned with those domains previously described in the Theoretical Domains Framework (TDF). We subsequently used TDF instead of Grounded Theory for the remainder of the study. TDF, developed by Michie et al. and validated by others, incorporates a broad spectrum of individual and organizational theories to study and better understand the motivations and behaviors of both physicians and patients in a medical setting [26, 27]. TDF consists of 12 discrete domains, elucidates facilitators and barriers to behaviors, and can act as a framework for future interventions [28, 29]. TDF has been successfully applied to a wide range of clinical topics such as the appropriateness of diagnostic imaging for spine disorders, perceived barriers to the reporting of adverse drug events, and the promotion of adherence to guidelines for suspected viral encephalitis [28, 30, 31]. TDF, a literature review, and the research team’s previously published findings describing guideline-discordant care informed the interview guides.

### Participants

Participants included veterans diagnosed with prostate cancer within 6 months prior to initial mail contact and physicians who reported caring for prostate cancer patients in the preceding 6 months. Three VAMCs were selected based on two criteria: (1) volume of prostate cancer patients (in order to ensure a sufficient patient sample) and (2) diversity of their rates of inappropriate and appropriate imaging (high, middle and low). We determined a site specific rate of appropriate and inappropriate prostate cancer imaging for each VAMC [21]. Sites classified as high had an average inappropriate imaging rate of 83 %, sites classified as middle had an average inappropriate imaging rate of 35 %, and sites classified as low had an average inappropriate imaging rate of 17 %. The median average yearly number of incident prostate cancer patients at the high sites was 119 (range, 44–380), the middle sites was 146 (range, 83–444) and at the low sites was 132 (range, 45–307). The goal of including sites across a variety of inappropriate imaging rates was to elicit a range of opinions on prostate cancer imaging at VHA.

### Sampling

Across the three sites, 22 patients and 17 physicians were interviewed (39 total) between September 2014 and July 2015. A total of 96 patients and 25 physicians were invited to participate in the semi-structured interviews (23 % of eligible patients and 68 % of eligible physicians participated). Among the 22 patients who participated in the interviews, 32 % were from the high site, 32 % from the middle site, and 36 % from the low site. Sixty-percent of the 17 physicians interviewed were from the high site, 23 % were from the middle site, and 17 % were from the low site. Patients with newly diagnosed prostate cancer were identified through a query of the VHA OncoTrax data in the VA Informatics and Computing Infrastructure (VINCI) database (determined with International Classification of Diseases, Ninth Revision, Clinical Modification diagnostic code 185). Patients with a history of prior malignancy, those aged over 85 years, and those who did not have data on PSA, clinical stage, or Gleason score were excluded. The study team contacted all eligible patients via mail and subsequent follow-up telephone calls; physicians were invited to participate via email. Patients were compensated $40 for their participation; per VHA research guidelines, physicians received no compensation for study participation. All patients were interviewed in-person by MS and ES. Interviews lasted an average of 31 min (range, 16—53). Physician interviews were either conducted in person or over the telephone, lasting an average of 45 min (range, 24–58) by DM and ES. Enrollment was concluded after achieving thematic saturation, the point at which the range of ideas has been elicited and subsequent interviews do not uncover new information [32].

### Data analysis

All interviews were audio-recorded and transcribed verbatim. Transcripts were entered into NVivo 8 qualitative software to facilitate data management and analysis. Three researchers (DM, ES, CC) independently reviewed transcripts to develop an initial codebook based on the TDF. MS, a medical anthropologist, provided a critique of the analysis and examined the coding to ensure a robust and defensible classification of the data into relevant domains. Two researchers (DM and ES) independently coded each transcript, modified the codebooks as themes emerged, and met to discuss and reconcile discrepancies until a final coded transcript was agreed upon. Disagreements around codes, themes, and subthemes were resolved by discussion among the team members (DM, ES, and CC) and reference to the original transcripts. There were a total of 34 codes in the physician codebook mapped to 10 TDF domains and 24 codes in the patient codebook mapped to 8 TDF domains. Emerging themes were further organized and analyzed using descriptive matrix analyses wherein the range of responses related to each theme was visually displayed [33]. After coding and analysis of the interviews, we identified the most applicable TDF domains (Table 1).

**Table 1:** The domains of the Theoretical Domains Framework most applicable to prostate cancer imaging, as determined from analysis of semi-structured interviews of patients and physicians

Relevant physician domains	Relevant patient domains
• Knowledge	• Knowledge
• Beliefs about consequences	• Beliefs about capabilities
• Beliefs about capabilities	• Emotion
• Social influences/norms	• Goals
• Environmental context and resources	

## Results

Each quote is followed by a designation (H: high rate of inappropriate imaging, M: middle rate of inappropriate imaging, L: low rate of inappropriate imaging) indicating the site at which the interview took place.

### Patients

#### Characteristics of patients

We interviewed 22 patients from three VAMCs. Eight patients (36 %) were African American, 12 (56 %) were white, one (5 %) was Asian, and one patient (5 %) did not identify his race. The median patient age was 69 (range 52–79). The veterans in our study population served in all four branches of the US Armed Forces for a median of 4 years (range 1.5–20). Half of the participants had undergone imaging to stage their prostate cancer (Table 2).

**Table 2:** Patient demographic information (*n* = 22)

Age, median (range)	69 (52–79)
Race	
White	12 (54.5 %)
Black	8 (36.4 %)
Asian	1 (4.5 %)
Unknown	1 (4.5 %)
PSA, median (range)	7.0 (2.8–37)
Gleason score	
≤6	6 (27.3 %)
>6	16 (72.7 %)
Imaging	
CT scan	11 (50.0 %)
Bone scan	3 (13.6 %)
No imaging	11 (50.0 %)
Treatment type	
Prostatectomy	7 (31.8 %)
Radiotherapy	7 (31.8 %)
Expectant management	6 (27.3 %)
Other	2 (9.1 %)

#### Patient findings: Key themes identified within relevant domains

Key themes emerging from the patient interviews were categorized within four domains: *goals*, *knowledge*, *beliefs about capabilities*, and *emotion.*

#### Domain: Goals

Patients were primarily interested in the treatment and outcomes of their prostate cancer. No patients reported specifically requesting an imaging evaluation. Patients’ attention was often so focused on treatment that many did not remember whether they had received imaging at all. Therefore, it was difficult for the interviewer to elicit specific responses about imaging because concerns regarding treatment and outcomes dominated the interview. Patients did not discuss the association between imaging results and subsequent choice of treatment.

I just was more concerned about what was going to happen after surgery and how I was going to deal with the recovery part of that. [H]

I really didn’t have any [expectations from my provider] really, you know. Just get me, you know, healthy, keep me healthy, you know, tell me what to do. [M]

#### Domain: Knowledge

Patients demonstrated limited knowledge about imaging, both as a general subject and regarding how imaging related to their care. The majority of patients could remember neither whether they had imaging tests nor the names of the tests they received prior to deciding on treatment. Most patients did not know why they had imaging, but some said that it was “to check if their cancer spread.” None of the twenty-two patients interviewed had any knowledge of any prostate cancer imaging guideline nor did they have any knowledge of the Choosing Wisely Campaign.

As the patients explained:

I don’t remember what they did. It seems like they did an x-ray, but I’m not sure… [L]

I just got in there and I had the MRI. And I don’t even know really what that was for. [H]

#### Domain: Beliefs about capabilities

Universally, patients stated that they trusted their doctor to make decisions about their care, especially related to imaging. Some patients reported curiosity leading them to investigate prostate cancer management on their own, but their inquiries primarily related to treatment options, not imaging. Other patients relied solely on their physician’s guidance. As one patient described:

I trust my doctor and he said this is the best course of action, I have that faith in him. No matter what anybody else tells me I’m going to do what my doctor told me. [H]

In addition to online research, many patients indicated that they consulted with friends or family regarding treatment, but none had consulted anyone regarding imaging specifically. One participant stated that his family or loved ones “appreciated” that the doctor ordered the test, but no patient stated that he had asked for imaging directly.

#### Domain: Emotion

The patients did not report concern with the process of imaging, which was commonly described as a routine part of prostate cancer care, nor the results of the tests. One patient recalls his emotional and fearful reaction to cancer outweighing any feelings about imaging.

I was so nervous. They had the big word that started with a C, that can dilute your memory. I don’t recall, off the top of my head, being told that I should get certain type of traditional testing. [H]

A few patients were mildly concerned about radiation or stated that they try to avoid it, but none described a sense of unease about exposure to radiation.

Going back to my history, I was guarding nuclear weapons. I got trained in radiation. And I try and avoid any kind of, as much radiation exposure as I can.” [L]

They’re [x-rays] much, much safer now than they were then. They use just a fraction of the power. [L]

### Physicians

#### Characteristics of physicians

We interviewed 17 physicians, all urologists practicing in VHA. The physicians were a median of 11 years out from completing residency training (range 1–31). Thirteen physicians (76%) were board certified in urology, and a number of them were fellowship-trained in subspecialties such as urologic oncology, male infertility and microsurgery, minimally invasive oncology, renal physiology, and health services research. The physicians reported seeing a median of 6 (range 2–12) incident prostate cancer cases per month (Table 3).

**Table 3:** Demographic information of physicians interviewed (*n* = 17)

Median years from residency (range)	11 (1–31)
Gender	
Male	1 (6 %)
Female	16 (94 %)
Race	
White	11 (65 %)
Black	2 (12 %)
Asian/Pacific Islander	4 (24 %)
Board certified (%)	13 (76 %)
Sample of fellowship areas	• Urologic oncology • Male infertility and microsurgery • Minimally invasive oncology • Renal physiology • Health services research
Median number of incident prostate cancer cases per month^a^	6
Range of incident prostate cancer cases per month^a^	2–12

^a^As reported by the physician

#### Physician findings: Key themes identified within relevant domains

Key themes emerging from the physician interviews were categorized within five domains: *knowledge*, *beliefs about consequences*, *beliefs about capabilities*, *social influences*, and *environmental context.*

#### Domain: Knowledge

All physicians felt knowledgeable about the existence of various published prostate cancer imaging guidelines, whether those promulgated by the National Comprehensive Cancer Network (NCCN), the European Association of Urology (EAU), or the American Urological Association (AUA), and how to access them (online, mobile app, or a print out) but their self-reported knowledge of the guidelines’ specific recommendations varied.

At our VHA myself and most of the other people that run our clinic have the NCCN guidelines App and AUA App on our phone. [H]

The average urologist in practice in the United States is aware the guidelines exist, is generally aware of what the guidelines say you should do but probably doesn’t keep up with the down and dirty details of the guidelines as they evolve. [L]

Additionally, while many physicians stated that they “believe in and understand the guidelines,” they also acknowledged practicing outside of the guidelines to varying degrees. The majority of the physicians who imaged outside of guideline recommendations stated that one reason for their behavior was their perception of a “gray area” within the guidelines.

I would say I have – I have firm cutoffs for who doesn't need imaging and who does need imaging, but there's a – there's a gray area in between and so the firm cutoff for who doesn't need imaging is essentially going to be anyone who has low risk prostate cancer. [H]

Therefore, even if physicians espouse extensive knowledge of the current guidelines, they still express uncertainty regarding the appropriateness of imaging within at least a subset of patients.

#### Domain: Beliefs about consequences

Many physicians stated that one reason that they perform guideline-discordant imaging is to assuage their own fears of missing a clinically significant, potentially unrelated diagnosis. The majority of physicians felt the dose of radiation associated with prostate cancer imaging was safe and did not affect their decision. When physicians did discuss the downsides or harms of over imaging, it was often regarding the ramifications of incidental findings.

I think they're just worried because they don't want to miss something. They don't want …to deny the patient the proper treatment because they missed something and a year later they found mets [metastatic disease]. [H]

Usually the concern about metastatic spread often trumps the concern about radiation. [M]

Um, because what ends up happening with over imaging is that you find things that aren't really clinically important. [H]

Many physicians reported that fear of litigation affects their own imaging practices and an extra, albeit inappropriate, test can provide “comfort” (i.e., peace of mind). On the other hand, some physicians reported that guidelines can provide some “comfort” (in the sense of protection from litigation) in ordering imaging appropriately.

Medical malpractice is alive and well, and I think it probably impacts decision making more than most clinicians will admit to. [H]

The idea that, my God, if I miss it I’m going to get sued. So people try to throw as much imaging as they can to kind of deflect the level of responsibility from themselves. [H]

#### Domain: Beliefs about capabilities

Many physicians vacillated during the interview about whether they follow the guidelines, their intuition, or their own personal protocol. While most endorsed a belief in the guidelines, many physicians felt that experience or intuition should take precedence over guidelines, especially when the patient is in the “gray area.” Less time since completion of residency training was mentioned by several physicians as an indicator that they would be more likely to follow guidelines.

Well if there was some clinical factor that or some clinical suspicion that, you know, the guidelines are sort of intended to direct us and I mean I think it would be very, very infrequent that we’d veer from the guideline based on just my judgment alone. [L]

Over or underutilization of imaging depends on the provider’s level of training, how far they are from residency, whether they’re practicing alone, you know. [L]

Physicians with more experience and more time since completion of residency training stated that they are more likely to follow their intuition or rely on their experience.

I understand the guidelines and I know them but I’m, I’m going to go against them for this particular reason and that’s why we go to medical school, to have our own opinion on certain things.” [H]

“I routinely obtain imaging on all patients who have been recently diagnosed with prostate cancer.” [H]

While most physicians stated that they would always image patients if it was indicated in the guidelines, a few physicians reported that they might forgo even guideline-recommended imaging if certain patient factors were present.

I may not do imaging [even when it is guideline indicated] for extreme elderly patients or patients that outright refuse any form of treatment. [H]

No, I can’t think of any reason why I wouldn’t [image when it was guideline indicated] for a patient with high risk disease. [H]

#### Domain: Social influences/norms

Most participants in the middle and high imaging hospitals said that they do not have department-wide standards and their colleagues do not routinely discuss imaging use.

We don’t have a rigid department-wide guideline for this. That much I can assure you. [H]

On the contrary, some physicians indicated that being a part of an academic institution, regular discussions with their colleagues and a general consensus to practice guideline-concordant care (in place at the low imaging hospital) assisted in practicing high quality care and guideline concordance.

I’ve got the benefit of being here with a large group of colleagues at a major university, so I have the luxury of a lot of smart people around me to keep me up to date but not all urologists at other VHAs have that [L]

Within all three VHA hospitals, the physicians identified a colleague in their department who influenced others’ imaging behaviors whether it was to image more or to image within the guidelines.

He does our oncology surgery and he wants to see a CT scan if they go to surgery. [H]

They tend to follow the practice of the supervising MD and so when you have mid-levels in the clinic with the particular MD who is ordering the studies for intermediate and low grade disease we had mid-levels also doing the same thing so we have had to deal with that [M]

#### Domain: Environmental context and resources

All interviewed physicians were affiliated with both an academic institution and VHA. Many commented on the differences between practice norms in VHA and those of their private or university clinics. Physicians compared VHA to private or ‘outside care’ in several areas including: differences in the patient population, quality of imaging equipment, quality of the radiographic interpretation, and financial incentives for providers (which are not directly present in VHA).

Yes absolutely yes there is nothing, there is no motivation here for that [imaging], which is actually one of the nice things about the VHA. I think that we can really focus on the guidelines and focus on giving really stream lined top of the level care based on best evidence. [M]

I have noticed that there is a … seems to be a higher rate of positive findings on imaging at the VHA that are not clinically relevant. And so I have been dismayed at the quality of our radiologists. [M]

Additionally, physicians stated that inappropriate imaging takes away resources from other important areas within the department or hospital.

We were getting bone scans on every patient diagnosed with prostate cancer and that changed when somebody woke up and said this is not cost effective. [H]

If you’re doing something because it’s inexpensive but ineffective it’s still expensive. [H]

Some physicians stated that their patients who are veterans are less assertive, tend to “trust their doctor,” and therefore are less likely to ask for additional tests than patients seen in their private practice. VHA physicians stated that these phenomena make it less likely that VHA patients will research the extent of their disease, predictors of treatment outcomes, or imaging options.

They [VHA patients] typically will accept your answers and defer to you because they have an innate trust and respect for doctors. [M]

The veterans though as a group I think are less likely to push for it [imaging], ask for it or be disappointed if you’re telling them it’s not going to be done. [M]

#### Domain: Miscellaneous; Construct: Intervention

When asked about a recommendation to improve guideline-concordant imaging, most physicians enthusiastically supported the idea of such an intervention and suggested the creation and implementation of a Computerized Patient Record System (CPRS) alert or some other electronic medical record (EMR)-based strategy to automatically communicate to them when they order guideline-discordant prostate cancer imaging. CPRS is a centralized EMR application within VHA that allows physicians to enter, review, and update patient information [34]. CPRS is also designed to assist with clinical-decision making [34]. Many physicians reported that they are accustomed to these types of reminders but warned against CPRS alert fatigue. Some participants did not explicitly mention an EMR-based intervention but all recommended education in some capacity as an important component of a behavioral intervention.

There could be things embedded in CPRS to help guide the practitioner in making these decisions. That’s unique to the VHA though because not every urology practice has access to electronic medical records that functions like CPRS. [L]

From an intervention standpoint provider education would seem to me to be the easiest. [L]

### Imaging variation across medical centers

There were stark differences in the physician responses from the three participating VAMCs (Table 4). Physicians from the high imaging utilization site tended to rely more on intuition or professional experience rather than guidelines, while physicians from the low imaging utilization site stated that they almost always image within guidelines except for rare cases or those which fell in the “gray area;” the middle imaging utilization site fell somewhere in between. The high site identified one colleague (whose practices nobody challenged) who routinely images all low-risk patients. The low utilization site’s chief of urology sits on a quality improvement committee and discusses guideline adherence with the department regularly. Physician knowledge of the guidelines varied (most starkly at the high imaging utilization site) based on their level of involvement in research, either their own research or reading research publications (more involvement with research was associated with greater professed understanding of the guidelines). The low imaging utilization site reported that attending conferences and being affiliated with an academic institution kept all physicians up to date on guidelines, while the intermediate imaging utilization site gave a variety of inconsistent responses. As one physician noted:

**Table 4:** Differences in the three medical centers—illustrated by quotes

Themes	Low rates of inappropriate imaging	Middle rates of inappropriate imaging	High rates of inappropriate imaging
Knowledge of guidelines	“Obviously everyone does things differently, but I’m … I take a very simplistic approach to it. Where I essentially follow the NCCN Guidelines.”	“I use them sometimes. Guidelines as a guideline but I tend to be more cautious, or shall we say more aggressive in terms of diagnostic testing.”	“I think as clinicians we can’t just follow a cookbook recipe.”
Intuition vs. guidelines	“When I look at something, be it a guideline or an article, I say mm – here’s absolute proof that getting that study doesn’t impact the outcome a bit, that is evidence.”	“So guidelines are just that they guide your care they don’t prescribe the care for you.”	“So I may be capturing a little bit more and exposing a few more people to imaging but that’s my internal guidelines.”
Colleagues’ imaging habits	“They’re doing things appropriately here but at other VA’s I’ve seen anyone with a diagnosis of prostate cancer will get a, a kneejerk bone scan and CT scan.”	“We have spoken with this particular individual [image avid colleague] but we found that this was his practice preference and he really wanted to stick with it although he understood what the guidelines were.”	“We’re all part of the referral process to him and so we all know what he feels is the next step [always obtain a CT scan].”
Tendency to question colleagues or discuss guidelines	“I think their answers would be very similar to mine. We all have a very similar practice pattern since we’re all practicing with the same university and VA. We try to follow, you know, guideline concordant care across all the different types of cancer we treat.”	“And so we’ve had a number of patients with Gleason 6 disease and somebody ordered a bone scan on them and now we have to figure out what these questionable areas of uptake mean. And we spend a lot of time trying to discuss these findings to figure out what to do with them.”	“We have a fellowship – a memorial trained oncology guy and to tell you the truth, to argue with him [about imaging practices] is pretty arrogant or discourteous.”

If you go to the VHA or any other provider in the U.S. with the same diagnosis of prostate cancer, you may get … totally different imaging. [M]

## Discussion

In this theory-based, qualitative exploration of prostate cancer imaging behavior, we found a disconnect between the value that physicians place on imaging and the value that patients place on it. Most patients’ knowledge about whether or not they had imaging was limited. Patients tended to trust their doctor to make decisions about imaging. Most did not fear exposure to radiation, but a few stated that they would rather avoid it. It is not surprising that patients were focused on treatment options and outcomes, which involve the greatest risk to the patient’s well-being and offer the promise of prostate cancer cure. All physicians felt knowledgeable about prostate cancer imaging guidelines but many reported imaging outside of guidelines out of fear of “missing something clinically significant“ or “fear of litigation.” Physicians who were further removed from residency training reported relying on their experience more than on guidelines, in contrast to physicians who more recently completed residency training. Additionally, one imaging avid colleague (a surgeon requiring all of his/her patients obtain imaging prior to surgery) was described as influencing prostate cancer imaging utilization across an entire VAMC. All physicians believed that a behavior change intervention was a worthwhile undertaking and suggested a program of physician education in some form; many suggested an EMR-based intervention such as a pop up alert in CPRS. Our findings suggest that physicians, not prostate cancer patients, are the primary drivers of prostate cancer imaging within the VHA.

Our findings are consistent with the existing literature on barriers and facilitators to guideline adherence in broader clinical settings outside prostate cancer. The themes of fear of missed pathology or fear of litigation have been salient in other qualitative studies. Providers have reported that they would rather manage the consequences of guideline-discordant imaging overuse than be burdened by fear of missing a diagnosis or getting sued [35–37]. We found that physicians further from residency training report relying on personal intuition more so than do recent graduates. Similarly, previous literature found senior ICU physicians report a preference for relying on personal experience rather than objective protocols (which they felt limited their autonomy), while more junior physicians were more likely to rely on guidelines [36]. Existing qualitative findings also reveal that clinicians in a primary care setting do not adhere to strictly to published guidelines, instead adopting heuristics developed from personal experiences and interactions with colleagues and patients [38]. Similar to our findings that colleagues influence prostate cancer imaging behavior, previous literature in the ICU setting has reported the effects of social pressure on guideline utilization [39].

Using a qualitative approach to understand the barriers and facilitators to guideline-concordant prostate cancer imaging is a critical step to inform design of an intervention to encourage guideline-concordant behavior. Two prior efforts to implement guideline-concordant prostate cancer imaging did so without the benefit of such qualitative data and demonstrated mixed results. The National Prostate Cancer Register (NPCR) of Sweden [40] established an audit and feedback program in which national-level data were used to generate local, hospital-level reports of the frequency of inappropriate imaging for low-risk prostate cancer patients. Additionally, the NPCR educated physicians by presenting them with the most recent versions of imaging guidelines and important literature and stressed the importance of reducing inappropriate imaging as a national priority [8]. In another quality improvement intervention, Miller et al. report on a similar strategy employing audit and feedback combined with physician education implemented within a Michigan State quality improvement consortium [4, 22]. In both cases, inappropriate imaging of low-risk patients declined significantly but so did appropriate imaging among high-risk patients [41].

These prior studies reporting quality improvement efforts based on audit and feedback and physician education, strategies supported by two landmark Cochrane reviews, might form the basis of an effective multi-level intervention to reduce inappropriate imaging [42, 43]. Our data additionally suggest physician interest in an EMR-based decision support tool. In the implementation science literature, there is strong evidence supporting this intervention strategy, particularly as it relates to advanced imaging [44, 45]. Computer-based audit and feedback systems have proven to generate moderate improvements in guideline adherence among primary care and ICU clinicians [39, 46]. However, practical challenges to the fidelity of such an intervention might limit its effectiveness. Efforts to implement an EMR-based intervention are likely to depend upon context and require tailoring to the situational factors of the implementation setting [47, 48]. The expression of interest in an EMR-based intervention by the physicians interviewed in this study suggests optimal morale for uptake of this strategy.

We found physicians had strong and altruistic, though sometimes misguided, rationales for performing guideline-discordant imaging in patients with low-risk disease. Physicians for the most part felt that they always image high-risk patients. This fraction of high-risk patients not undergoing imaging might fall within a gray area for physicians who use personal imaging heuristics, where the physicians might not deem imaging to be necessary. While data against imaging overuse clearly demonstrate its low utility among low-risk patients, data supporting a mandate for imaging in high-risk patients are less compelling [49–52]. The low-risk category may be variably-defined but would always include a group of patients with Gleason less than 7, stage T2 or less and PSA less than 10 [10]; this group represents the overwhelming majority of men with incident prostate cancer [14]. Imaging in this cohort would be inappropriate regardless of the guideline a physician might use to determine the need for imaging, but this gray area defining high-risk might create confusion over which of the smaller group of patients with higher-risk features need imaging. While the gray areas apply to very few patients, physicians in our study seemed to bring them up more frequently than might otherwise be expected, perhaps suggesting that even minor discrepancies between various versions of imaging guidelines might confuse their end users and make all guidelines less accessible. Therefore, an intervention focused solely on reducing imaging might have decreased the propensity to order imaging for patients perceived to be in the gray area. Interventions seeking to decrease inappropriate imaging among low-risk patients and simultaneously maintain or improve appropriate imaging among high-risk patients might focus on clearly defining patients who need imaging (perhaps by defining a clear threshold risk/benefit ratio for imaging) and actively encouraging the practice there [53].

Employing the TDF is an important step to developing such an intervention because it allows exploration of a common set of barriers and facilitators across studies within a theoretical framework. A 2012 study explored the beliefs of chiropractors about adherence to evidence-based recommendations for spine radiography for uncomplicated back pain. In this study, five domains were identified as relevant in the *beliefs about consequences*, *beliefs about capabilities*, *social professional role and identity*, *social influences*, and *knowledge* [28]. Our study confirmed the importance of all five of these domains and identified an additional domain, *the environmental context*. In our study, physicians reported that the radiologist interpreting the image and the quality of the imaging equipment differ between the VHA and private practice, as do the preferences of patients. Physicians also stated that VHA patients differ from private practice patients in terms of knowledge of or tendency to request imaging.

Some physicians reported that patients “appreciate” imaging. Providing patient centered care is often a cornerstone of best practices, especially in VHA [54]. The physician is therefore presented with a dilemma when a low-risk prostate cancer patient requests imaging outside of suggested guidelines. Such an encounter presents an opportunity for physicians to educate their patients about best evidence and national guidelines (e.g., Choosing Wisely). This discussion may lead to the patient accepting the guideline recommendation or continuing to wish for guideline-discordant practices. While such a choice is, by definition, “guideline-discordant,” many experts in evidence-based medicine would still support the decision [55]. In the current study, patient preferences were not reported to be a key barrier to guideline-concordant imaging, but in future situations where patient preferences were driving the inappropriate testing, a patient educational component might be considered as part of a behavioral intervention to reduce guideline-discordant imaging.

Additionally, the NCCN, EUA, and AUA committees charged with creating and revising imaging guidelines should consider clarifying how to address these “gray area” patients mentioned by many physicians. There were many contradictions, and responses sometimes vacillated within an individual interview. Several times, physicians would state that they attempt to follow the guidelines but later would describe how they image outside of guidelines for various reasons. There are multiple factors that contribute to physicians’ decisions to image, and based on the interview questions, different and even contradictory responses were elicited. An intervention addressing each of the complex factors elicited in this study would make the greatest impact, one such part of such an intervention might simplify the discrimination between high and low-risk patients [29].

### Study limitations

This study was designed to generate deeper knowledge and understanding of patient and physician attitudes and beliefs in a specific context. Our findings may not be generalizable to other settings or other populations, despite reaching theoretical saturation [32, 56]. Additionally, physician knowledge was not explicitly tested so knowledge about imaging guidelines was self-reported; neither physician-specific nor patient-specific imaging behavior was recorded. However, it was important while developing rapport between interviewer and study subject, not to appear to be evaluating the participant’s knowledge. Lastly, we did not include radiologists, medical oncologists, or radiation oncologists in our interviews. These potential stakeholders may also have provided important perspectives. However, urologists are most often responsible for diagnosing patients with prostate cancer because they are the ones to first evaluate patients with elevated PSA or symptoms suggestive of prostate cancer and are the ones subsequently to stage the disease prior to treatment. Therefore, urologists are most directly responsible for staging prostate cancer and ordering imaging tests as a part of that process.

## Conclusions

The results of this TDF-based study of barriers and facilitators to guideline-concordant prostate cancer imaging suggest physicians’ attitudes are the primary driver of utilization. Patients tended to trust their doctor to make decisions about appropriate tests and reported little interest in disease staging as compared to treatment. This combination of attitudes and behaviors suggests targeting physicians might be the most effective strategy for improving guideline-concordant prostate cancer imaging. Using TDF to classify and analyze qualitative data about this challenging clinical dilemma not only provides a theoretical framework for understanding these barriers and facilitators but also an algorithm to identify a set of intervention strategies to address them [29]. Such behavior change strategies might involve creating EMR-based decision supports, as well as strategies to address concerns about missed pathology and provide protection against litigation for physicians complying with guideline recommendations. Other interventions such as audit and feedback and academic detailing, which address other domains with complementary intervention functions, might be bundled with these to achieve optimal effectiveness, since they have demonstrated effectiveness in prior quality improvement initiatives and have strong theoretical rationales for their use [16, 29, 41, 53, 57–60]. This study represents the first step in a theory-based pathway to identify and overcome the challenges to guideline-concordant prostate cancer imaging [25, 29, 41]. These qualitative data and our current approach to improve the quality of care for men with prostate cancer may serve as a roadmap to researchers as they work to improve the quality of cancer care and imaging appropriateness in other settings.

## Abbreviations

ASCO: American Society of Clinical Oncology
AUA: American Urological Association
CPRS: Computerized Patient Record System
CT: Computerized tomography
VA: Department of Veterans Affairs
EMR: Electronic medical record
EAU: European Association of Urology
MRI: Magnetic resonance imaging
NCCN: National Comprehensive Cancer Network
NPCR: National Prostate Cancer Register of Sweden
PSA: Prostate-specific antigen
TDF: Theoretical Domains Framework
VINCI: VA Informatics and Computing Infrastructure
VAMCs: VA Medical Centers
VHA: Veterans Health Administration
